# Renal Phosphate Handling: Independent Effects of Circulating FGF23, PTH, and Calcium

**DOI:** 10.1002/jbm4.10437

**Published:** 2020-12-09

**Authors:** Malachi J McKenna, Rachel K Crowley, Patrick J Twomey, Mark T Kilbane

**Affiliations:** ^1^ UCD School of Medicine University College Dublin Dublin Ireland; ^2^ Department of Clinical Chemistry St. Vincent's University Hospital Dublin Ireland; ^3^ Department of Endocrinology St. Vincent's University Hospital Dublin Ireland

**Keywords:** FIBROBLAST GROWTH FACTOR 23, HYPERPARATHYROIDISM, HYPOPARATHYROIDISM, PARATHYROID HORMONE, X‐LINKED HYPOPHOSPHATEMIA

## Abstract

Excess fibroblast growth factor 23 (FGF23), excess PTH, and an increase in extracellular calcium cause hypophosphatemia by lowering the maximum renal phosphate reabsorption threshold (TmP/GFR). We recently reported two cases of X‐linked hypophosphatemia (XLH) with severe tertiary hyperparathyroidism who had normalization of TmP/GFR upon being rendered hypoparathyroid following total parathyroidectomy, despite marked excess in both C‐terminal FGF23 (cFGF23) and intact FGF23 (iFGF23). We explored the effects of FGF23, PTH, and calcium on TmP/GFR in a cross‐sectional study (*n* = 74) across a spectrum of clinical cases with abnormalities in TmP/GFR, PTH, and FGF23. This comprised three groups: FGF23‐dependent hypophosphatemia (*n* = 27), hypoparathyroidism (HOPT; *n* = 17), and chronic kidney disease (*n* = 30). Measurements included TmP/GFR, cFGF23, PTH, ionized calcium, vitamin D metabolites, and bone turnover markers. The combined effect of cFGF23, PTH, and ionized calcium on TmP/GFR was modeled using hierarchical multiple regression and was probed by moderation analysis with PROCESS. Modeling analysis showed independent effects on TmP/GFR by cFGF23, PTH, and ionized calcium in conjunction with a weak but significant effect of the interaction term for PTH and FGF23; probing showed that the effect was most prominent during PTH deficiency. Teriparatide 20 μg daily was self‐administered for 28 days by one case of X‐linked hypophosphatemia with hypoparathyroidism (XLH‐HOPT) to assess the response of TmP/GFR, cFGF23, iFGF23, nephrogenous cyclic adenosine monophosphate (NcAMP), vitamin D metabolites, and bone turnover markers. After 28 days, TmP/GFR was lowered from 1.10 mmol/L to 0.48 mmol/L; this was accompanied by increases in NcAMP, ionized calcium, and bone turnover markers. In conclusion, the effect of FGF23 excess on TmP/GFR is altered by PTH such that the effect is ameliorated by hypoparathyroidism and the effect is augmented by hyperparathyroidism. © 2020 The Authors. *JBMR Plus* published by Wiley Periodicals LLC. on behalf of American Society for Bone and Mineral Research.

## Introduction

The two major hormonal determinants of phosphate homeostasis, fibroblast growth factor 23 (FGF23) and PTH, regulate renal phosphate excretion by reducing the expression of the same sodium‐dependent phosphate cotransporters, NaPi‐2a and NaPi‐2c, on the luminal membrane of the proximal renal tubule.^(^ ^1^, ^2^ ^)^ FGF23 excess causes chronic hypophosphatemia by lowering the maximum renal tubular phosphate reabsorption rate per volume of glomerular filtrate (TmP/GFR) with a resultant severe mineralization defect that manifests as rickets and osteomalacia in childhood and as osteomalacia in adults.^(^ ^3^, ^4^ ^)^ Disorders of sustained FGF23 excess are rare, but they encompass both congenital disorders such as XLH and acquired diseases such as tumor‐induced osteomalacia (TIO).^(^ ^3^, ^5^ ^)^ PTH excess caused by primary hyperparathyroidism causes hypophosphatemia by lowering TmP/GFR, but unlike hypophosphatemic bone disease does not cause osteomalacia.^(^ ^6^, ^7^ ^)^ Secondary hyperparathyroidism with low TmP/GFR and hypophosphatemia as a consequence of vitamin D deficiency manifests as a high bone turnover state that culminates in osteomalacia when deficiency is severe and sustained.^(^ ^8^ ^)^

Intuitively, because both PTH and FGF23 act through the same sodium‐dependent phosphate cotransporters, PTH status could alter the magnitude of the effect of FGF23 excess on TmP/GFR. Such an effect was first found in patients with hypoparathyroidism. High C‐terminal FGF23 (cFGF23) did not normalize serum phosphate in the setting of low or undetectable PTH, suggesting that the phosphate lowering effect of FGF23 requires PTH.^(^ ^9^ ^)^ This effect was confirmed by administering cinacalcet to two cases of TIO.^(^ ^10^ ^)^ Cinacalcet decreased both calcium and PTH; phosphaturia was reduced and serum phosphate increased, despite an increase in cFGF23. After 9 months therapy, cinacalcet had ameliorated osteomalacia.^(^ ^10^ ^)^ Thus, the authors concluded that the phosphaturic effect of FGF23 excess in TIO is diminished by medically induced hypoparathyroidism.^(^ ^10^ ^)^ In support of this observation, it was determined in XLH cases with secondary hyperparathyroidism that the administration of paricalcitol, a vitamin D analog, compared with placebo, suppressed PTH and increased TmP/GFR despite an increase in intact FGF23 (iFGF23).^(^ ^11^ ^)^ Similarly, case reports of hypoparathyroidism postparathyroidectomy in both TIO and XLH normalized TmP/GFR, despite high cFGF23.^(^ ^12^, ^13^, ^14^ ^)^ This phenomenon, with hindsight, was evident in earlier case reports of XLH in the era prior to FGF23 discovery, even including the original publication by Albright and colleagues that reported normalization of serum phosphate after parathyroidectomy.^(^ ^15^, ^16^, ^17^ ^)^ The opposite effect is seen when there is PTH excess caused by secondary hyperparathyroidism in the setting of XLH that leads to further lowering of serum phosphate^(^ ^11^, ^18^ ^)^ and TmP/GFR.^(^ ^11^ ^)^

The potential for an independent effect of extracellular calcium on TmP/GFR must be considered based on earlier studies: Conflicting results about the effect of calcium on renal phosphate wasting in XLH have been reported. Increasing serum calcium in XLH by calcium infusions reduced renal phosphate wasting, which suggested an indirect effect consequent upon lowering of PTH by the calcium infusion.^(^ ^19^, ^20^ ^)^ The opposite effect of serum calcium was reported in a case of XLH and coincidental HOPT, whereby medical treatment that increased serum calcium also increased renal phosphate wasting. This suggests that in HOPT an increase in circulating calcium has a direct effect on the lowering the TmP/GFR.^(^ ^17^ ^)^

We reported two cases of XLH and hypoparathyroidism postthyroidectomy (XLH‐HOPT) with normal TmP/GFR and with exceedingly high FGF23, both cFGF23 and iFGF23, a finding that has not been reported previously, but both cases had chronic kidney disease.^(^ ^13^, ^14^ ^)^ Based on the findings of these two cases and on the observations in the literature outlined above, we sought to explore the independent effects of FGF23, PTH, and calcium on TmP/GFR. We studied three discrete patient groups with different characteristics but all with high cFGF23. The first group with FGF23‐dependent hypophosphatemia (FDH)—having low TmP/GFR, high cFGF23, normal renal function, and normal or mild PTH elevation—was recruited to assess the effect of elevated cFGF23 upon TmP/GFR in the presence of variable PTH activity. A second group with hypoparathyroidism (HOPT) was recruited to assess the range of cFGF23 and TmP/GFR in the setting of PTH deficiency. Because the two cases of XLH‐HOPT had chronic kidney disease (CKD),^(^ ^13^, ^14^ ^)^ a third group of patients with stable CKD was recruited to assess the confounding effect of CKD. In addition to the clinical groups, we included our two index cases of XLH‐HOPT who had a degree of CKD; thus, these two cases represent a small crossover group with elements in common with the other three groups. We also included a case of FGF23‐independent renal phosphate wasting caused by renal tubular acidosis (RTA) for comparison purposes. In one case of XLH‐HOPT, we explored the effect of short‐term recombinant human parathyroid hormone analog (rhPTH1‐34) on TmP/GFR.

## Patients and Methods

### Patients

All parts of the study were approved by the ethics committee at St. Vincent's University Hospital with patients providing informed consent. There were 77 participants that included three discrete patient groups: two cases of XLH‐HOPT and one case of RTA (the latter was included for reasons explained below). The principal group incorporated patients with FDH (*n* = 27) that included congenital hypophosphatemia (XLH, *n* = 22; autosomal dominant hypophosphatemic rickets, *n* = 2) and TIO (*n* = 3). The patients with congenital hypophosphatemia were all adult. Information on clinical findings, genetic mutations, biochemistry, and treatment were published recently.^(^ ^14^ ^)^ Secondary hyperparathyroidism (normal calcium with elevated PTH) was evident in 41%; none had tertiary hyperparathyroidism. The three adults with TIO have not been previously reported. All three presented with symptomatic osteomalacia, had low TmP/GFR, and had elevated cFGF23; none had secondary hyperparathyroidism. Two of them subsequently had successful excision of benign mesenchymal tumors. Data were collated prospectively in this FDH group as part of their care at our national referral center.^(^ ^14^ ^)^

A second group with HOPT (*n* = 17) was recruited from our clinical endocrinology service. The cause of HOPT was either postsurgical (*n* = 6) or idiopathic (*n* = 11). All presented with symptomatic hypocalcemia. All were treated with varying doses of alfacalcidol and calcium supplements; none were treated with recombinant human PTH. PTH was undetectable in 29%, low in 47%, and low‐normal in the remainder of patients. FGF23 was elevated in 71%, and TmP/GFR was elevated in 53%.

A convenience sample of patients with stable CKD (*n* = 30), who had an estimated glomerular filtration rate (eGFR) ranging from 12 to 54 mL/min, was recruited from our outpatient nephrology service. None of the CKD cases were on phosphate binders, and only two were taking low‐dose alfacalcidol. Secondary hyperparathyroidism was evident in 63%, but none had tertiary hyperparathyroidism. cFGF23 was elevated in 93%. TmP/GFR was low in 63%; no patients had hyperphosphatemia and one patient had hypophosphatemia. They were studied for three reasons: (i) because the two cases of XLH‐HOPT had CKD, (ii) because it is well‐known that both PTH and FGF23 increase with advancing CKD, and (iii) because renal function was considered to be a pertinent covariate for the modeling analysis.

The two cases of XLH‐HOPT had elective total parathyroidectomy for severe refractory tertiary hyperparathyroidism with declining renal function. Both cases needed alfacalcidol to prevent symptomatic hypocalcemia. Their details have been described previously.^(^ ^13^, ^14^ ^)^ A third clinical case was included of a man with FGF23‐independent hypophosphatemia caused by congenital distal RTA with sensorineural deafness (OMIM 267300 classification), who had compound heterozygote mutations of *ATP6V1B1* (c.687+1G>T with abnormal splicing of the transcript, and c.943C>T, P.R315X). He presented as an adult with Looser zones, low TmP/GFR, low cFGF23, and low iFGF23. This case was included as a contrast to the two cases of XLH‐HOPT with CKD because he had low TmP/GFR, low cFGF23, and low iFGF23 despite having CKD.

### Intervention study

One of the patients with XLH‐HOPT (case 1) self‐administered 20 μg rhPTH1‐34 (teriparatide) daily for 28 days. Fasting blood and urine tests were performed before injection and 4 hours after injection on both the first day and the final day.

### Laboratory studies

The following were measured in all patients according to methods previously described based on fasting samples of blood and of second morning‐void timed urine: TmP/GFR; cFGF23; PTH; creatinine; eGFR; ionized calcium; phosphate; 25‐hydroxyvitamin D (25OHD); 1,25 dihydroxyvitamin D [1,25(OH)_2_D]; bone‐specific alkaline phosphatase (bone ALP); N‐mid fragment osteocalcin [OC(1–43)]; total procollagen type I N‐terminal propeptide (PINP); C‐terminal telopeptide of type I collagen (CTX); and urine N‐terminal telopeptides of type I collagen (uNTX).^(^ ^14^ ^)^ A single sample was obtained from the HOPT group and CKD group. The results in many of the FDH cases and the three clinical cases are based on the average of multiple samples that had been taken prospectively over a variable time period as part of each patient's routine clinical care.^(^ ^14^ ^)^

Serum and urine samples for phosphate and creatinine were analyzed on a Roche Cobas 8000 automated chemistry system. TmP/GFR was calculated according to Walton and Bijvoet^(21)^ and Bijvoet.^(^ ^22^ ^)^ Serum measurements of 25OHD, PTH, CTX, PINP, and Oc were determined using the Roche Cobas e602 immunoassay system (Basel, Switzerland); samples for ionized calcium were analyzed on a Radiometer ABL800 FLEX blood gas analyzer. Bone ALP, cFGF23, and uNTX were analyzed using bone‐specific alkaline phosphatase—Ostase (IDS, Bolden Colliery, UK), Immutopics (Quidel, San Diego, CA), and Osteomark uNTX ELISA kits (Abbott, Chicago, IL), respectively. 1,25(OH)_2_D was measured on serum samples using either the immunodiagnostics radioimmunoassay (Bolden Colliery, UK) (2011 to July 2017) or the DiaSorin Liaison chemiluminescent assay (Stillwater, MN) (July 2017 onward). In the two cases of XLH‐HOPT and the case of RTA and sensorineural deafness, iFGF23 was measured in Norfolk and Norwich University Hospital (Norwich, England) using the Kainos ELISA method (Tokyo, Japan). Biochemical results were interpreted against reference intervals sourced from each assay manufacturer's published instructions for use. Reference intervals were independently tested for transference to our study population prior to the analysis of clinical samples according to the Clinical and Laboratory Standards Institute Guideline EP28‐A3C.^(^ ^23^ ^)^

For the patient who had the short trial of rhPTH1‐34, the following were measured before and after injection on day 1 and again on day 28: TmP/GFR; cFGF23; iFGF23; ionized calcium; bone turnover markers; cyclic adenosine monophosphate (cAMP) in serum and urine; soluble α‐Klotho; 25OHD; 1,25(OH)_2_D; and 24,25(OH)_2_D. Nephrogenous cAMP (NcAMP) was derived and normalized for GFR as follows: (i) Total cAMP = urine cAMP * (serum creatinine/urine creatinine * 1000), (ii) filtered cAMP = urine cAMP * (serum creatinine)/(serum cAMP * [urine creatinine *1000]), and (iii) NcAMP = total cAMP – filtered cAMP.^(^ ^24^, ^25^ ^)^ Serum concentrations of soluble α‐Klotho were assayed using an ELISA method (Immuno‐Biological Laboratories, Minneapolis, MN). Because there was not an in‐house verified reference interval, we chose a published reference interval.^(^ ^26^ ^)^ The vitamin D metabolites 25OHD; 1,25(OH)_2_D; and 24,25(OH)_2_D were measured by liquid chromatography–tandem mass spectrometry at Norfolk and Norwich University Hospital, and the following vitamin D metabolite ratios were derived: 25OHD:24,25(OH)_2_D and 1,25(OH)_2_D:24,25(OH)_2_D.^(^ ^27^ ^)^

### Statistical analysis

Descriptive statistics are presented as a number and percentage for categorical variables and as median and interquartile range for continuous variables. For samples with undetectable PTH (<0.6 pmol/L), the result was censored at 0.5 pmol/L. The PTH and cFGF23 distributions exhibited positive skewness, and thus were log‐transformed to remove this skewness and enable use of the robust parametric statistical tests. Differences in independent categorical variables were tested by chi‐square tests. Bivariate associations were investigated using Pearson product–moment correlation coefficient. Partial correlation was used to explore the relationships between ionized calcium, PTH, and TmP/GFR. Because the three groups were chosen for their separateness across a range of abnormalities, discriminant function analysis was performed to demonstrate the ability of three variables (TmP/GFR, log cFGF23, and log PTH) to predict membership of the three different groups. Differences between the three groups (FDH, HOPT, and CKD) were tested by one‐way between‐groups ANOVA. The Tukey honestly significant difference (HSD) test was used as the post hoc test of mean differences between the groups.

Using the combined data set from the three groups for pairwise analysis (*n* = 73; excluding one case with missing ionized calcium in the CKD group), the independent effects on TmP/GFR of FGF23, PTH, and ionized calcium were explored by hierarchical multiple regression analysis and by moderation analysis with PROCESS. Regarding potential covariates [age, sex, eGFR, and 1,25(OH0_2_D], only eGFR had a significant correlation with TmP/GFR (*r* = −0.235, *p* = .044); so eGFR was entered as a covariate in the modeling analyses. For the hierarchical multiple regression analysis, eGFR was entered for model 1; log PTH, log cFGF23, and ionized calcium were entered for model 2; and 3 two‐way interaction terms and one three‐way interaction term were entered for model 3: all being the product of mean centered variables. Moderation analysis used the conceptual model 1 of Hayes via the PROCESS procedure version 3.4.^(^ ^28^ ^)^ This is a single analysis following entry of TmP/GFR as the dependent variable, log cFGF23 as the predictor variable, log PTH as the moderator variable, and both ionized calcium and eGFR as covariates. An interaction term was calculated as part of this analysis as the product of log cFGF23 and log PTH. An advantage of this analysis is that it permits both probing and visualizing the conditional effect of FGF23 on TmP/GFR across a range of PTH values. Probing the interaction was approached in two ways: by a pick‐a‐point approach whereby the conditional effect of FGF23 on TmP/GFR was estimated at three PTH percentiles (16th, 50th, and 84th), and by the Johnson‐Neyman technique, which probed the effect across the entire continuum of PTH results, ranging from 0.5 pmol/L to 52.3 pmol/L.^(^ ^28^ ^)^ All statistics were performed using SPSS for Windows version 25 (IBM, Armonk, NY, USA). Statistical significance was set as *p* < .05. All graphs were created using GraphPad Prism version 8.4.2 (GraphPad Software, Inc., La Jolla, CA, USA).

## Results

### Preliminary statistics

Descriptive statistics and results of ANOVA are given in Table 1. Among the total group (*n* = 74), the missing data were as follows: 1,25(OH)_2_D (*n* = 5) and ionized calcium (*n* = 1). Among the three cases, the missing data were ionized calcium and 1,25(OH)_2_D in case 2. A three‐group discriminant function analysis simultaneously using three variables (TmP/GFR, log cFGF23 and log PTH) correctly classified 65 of 74 (87.8%) cases to their diagnostic group, as is visually apparent in the scatterplots (see below). TmP/GFR was lower in FDH than CKD (*p* < .001), which in turn was lower than HOPT (*p* < .001). Log cFGF23 was higher in CKD than both FDH (*p* = .021) and HOPT (*p* = .001). Log PTH was lowest in HOPT, intermediate in FDH, and highest in CKD. Ionized calcium was similar in FDH and CKD but was lower in HOPT (*p* < .001). 1,25(OH)_2_D was similar in FDH and HOPT (*p* = .806) but lower in CKD. Bone turnover markers were consistently lower in HOPT than FDH (*p* < .001 for bone ALP; *p* = .006 for PINP; *p* = .001 for CTX; *p* < .001 for NTX), except for OC(1–43) that was not significantly lower (*p* = 0.196). There was no consistent pattern in comparing bone turnover markers between CKD and both FDH and HOPT.

**Table 1:** Descriptive Statistics for the Three Groups With Results of Group Comparisons Using ANOVA and Results for the Three Cases

Variables	FDH (*n* = 27)	CKD (*n* = 30)	HOPT (*n* = 17)	*p* Values	Case 1	Case 2	Case 3	Reference intervals
Age, y	30 (20, 46)	59 (41, 71)	55 (33, 65)	<.001^a,b^	36	36	44	—
Sex, M:F	9:18	21:9	5:12	.005	F	F	M	—
TmP/GFR, mmol/L	0.44 (0.35, 0.56)	0.75 (0.65, 0.85)	1.42 (1.24, 1.68)	<.001^a,b,c^	1.10	0.92	0.51	0.81, 1.35
cFGF23, RU/mL	142 (96, 246)	264 (161, 434)	113 (93, 184)	.001^a,c^	10,015	4,310	23.1	<100
PTH, pmol/L	6.3 (4.7, 8.3)	9.5 (4.8, 16.0)	0.9 (<0.6, 2.0)	<.001^b,c^	<0.6	0.7	5.1	1.6, 6.9
eGFR, mL/min	116 (106, 127)	25 (19, 42)	76 (57, 85)	<.001^a,b,c^	36	26	43	—
Phosphate, mmol/L	0.60 (0.50, 0.69)	1.08 (1.24, 1.31)	1.39 (1.27, 1.59)	<.001^a,b,c^	1.35	1.16	0.85	0.80, 1.48
Ionized calcium, mmol/L	1.26 (1.25, 1.30)	1.28 (1.24, 1.28)	1.11 (0.99, 1.17)	<.001^b,c^	1.17	—	1.33	1.19, 1.35
25OHD, nmol/L	50 (42, 65)	44 (27, 67)	91 (57, 127)	<.001^b,c^	92.4	79.2	52.5	30, 125
1,25(OH)_2_D, pmol/L	94 (57, 130)	53 (43, 75)	82 (69, 105)	<.001^a,c^	68	—	124.8	55, 139
BAP, μg/L	31.8 (22.9, 60.1)	16.7 (13.4, 23.5)	13.4 (10.4, 17.2)	<.001^a,b^	11.9	16.4	86.4	2.9, 20.9
PINP, μg/L	84 (57, 152)	100 (52, 142)	32 (24, 82)	.002^b,c^	50.4	117	226.8	22, 96
OC(1–43), μg/L	31.8 (19.3, 42.5)	51.7 (25.8, 77.5)	15.4 (11.1, 24.2)	<.001^a,c^	22.8	30.6	83.3	11, 43
CTX, μg/L	0.80 (0.53, 1.14)	0.72 (0.56, 1.00)	0.25 (0.15, 0.64)	.002^b,c^	0.460	0.609	2.634	0.016, 0.584
NTX, nmol BCE/mmol Cr	65 (43, 96)	34 (2, 71)	25 (17, 52)	<.001^a,b^	25.8	42.4	195.5	14, 74
Ca:Cr, mmol/mmol	0.13 (0.06, 0.25)	0.05 (<0.04, 0.11)	0.28 (0.12, 0.45)	<.001^b,c^	0.49	0.35	0.44	0.07, 0.41

The three groups are FGF23‐dependent hypophosphatemia (FDH), chronic kidney disease (CKD), and hypoparathyroidism (HOPT). Group results are presented as median (interquartile range) or as frequency. FGF23 and PTH were log‐transformed prior to ANOVA. *P* values refer to chi‐square test for sex and to one‐way ANOVA for scale variables, with superscripts indicating significant post hoc comparisons using Tukey HSD: ^a^FDH vs CKD, ^b^FDH vs HOPT, and ^c^CKD vs HOPT. Case 1 and case 2 refer to the two patients with X‐linked hypophosphatemia with hypoparathyroidism. Case 3 refers to the patient with renal tubular acidosis and sensorineural deafness. 1,25(OH)_2_D = 1,25‐hydroxyvitamin D; 25OHD = 25‐hydroxyvitamin D; BAP = bone‐specific alkaline phosphatase; cFGF23 = C‐terminal FGF23; Ca = calcium; Cr = creatinine; CTX = C‐terminal telopeptide of type I collagen; eGFR, estimated glomerular filtration rate; OC(1–43) = N‐mid fragment osteocalcin; PINP = procollagen type I N propeptide; BCE = bone collagen equivalents; NTX = N‐terminal telopeptides of type I collagen; RU = relative unit; TmP/GFR = maximum renal tubular phosphate reabsorption rate per volume of glomerular filtrate.

### Correlation analyses

There were significant correlations between TmP/GFR, log FGF23, log PTH, and ionized (Table 2). Since ionized calcium was positively correlated with log PTH (*r =* .645, *p* < .001), partial correlation was used to explore the relationship between TmP/GFR, log PTH and ionized calcium. Partial correlation between ionized calcium and TmP/GFR, while controlling for log PTH, was still significant (*r =* −.563, *p* < .001) but lower than the zero‐order correlation (*r =* −.756, *p* < .001). Conversely, the partial correlation between log PTH and TmP/GFR, while controlling for ionized calcium, was still significant (*r =* −.404, *p* < .001) but lower than the zero‐order correlation (*r =* −.690, *p* < .001).

**Table 2:** Correlation Matrix

	TmP/GFR	Log cFGF23	Log PTH	Ionized calcium
TmP/GFR	1	−.373^*^	−.690^**^	−.756^**^
Log cFGF23	−.373^*^	1	.511^**^	.340^*^
Log PTH	−.690^**^	.511^**^	1	.645^**^
Ionized calcium	−.756^**^	.340^*^	.645^**^	1

*n* = 74 (except for ionized calcium, *n* = 73). cFGF23 = C‐terminal FGF23; TmP/GFR = maximum renal tubular phosphate reabsorption rate per volume of glomerular filtrate.

* *p* < .01.

** *p* < .001.

Regarding 1,25(OH)_2_D, there was no correlation with TmP/GFR (*r* = .043, *p* = .726); so it was not included in the modeling analysis. There were significant correlations with 1,25(OH)_2_D in keeping with expectations: such as negative correlation with log cFGF23 (*r* = −.562, *p* < .001) and a positive correlation with eGFR (*r* = .587, *p* < .001).

**Fig 1:**
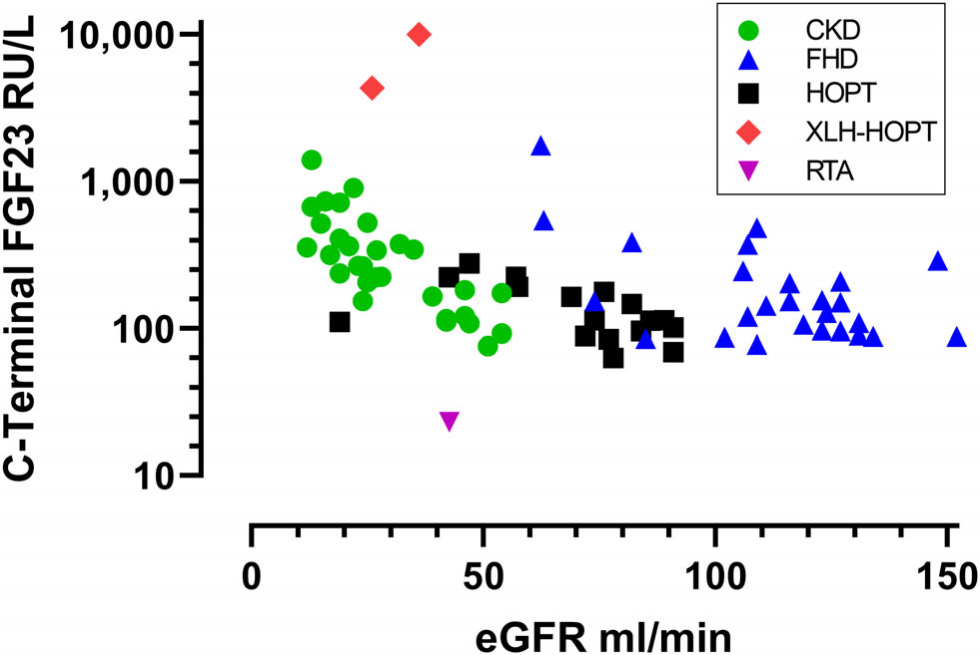
Scatterplot of log cFGF23 and eGFR that includes patients with FGF23‐dependent hypophosphatemia (blue triangles), hypoparathyroidism (black squares), chronic kidney disease (green circles), the two cases of XLH‐HOPT (red diamonds), and the case of renal tubular acidosis with sensorineural deafness (purple inverted triangle). There were significant negative correlations between eGFR and log cFGF23. They were for the composite of the three groups: *r =* −.508, *p* < .001; for CKD alone: *r =* −.798, *p* < .001; for FDH alone: *r =* −.543, *p* = .003; and for HOPT alone: *r =* −.520, *p* = .033. cFGF23 = C‐terminal FGF23; CKD = chronic kidney disease; eGFR = estimated glomerular filtration rate; FDH = FGF23‐dependent hypophosphatemia; XLH‐HOPT = X‐linked hypophosphatemia with hypoparathyroidism; RTA = renal tubular acidosis.

There were significant negative correlations between eGFR and log cFGF23 for the composite of the three groups and for each group individually (Fig. 1). The scatterplot for eGFR and log cFGF23 demonstrates that the two cases of XLH‐HOPT were outliers above all three groups (Fig. 1), and TmP/GFR was toward the upper end of that expected for degree of CKD (Table 1). The case of RTA was an outlier below the three groups. That case had the lowest cFGF23 at 23.1 RU/ml, which was the mean of six different results. In addition, iFGF23 was low at 28.6 pg/mL (reference interval, 33–110 pg/mL).

**Fig 2:**
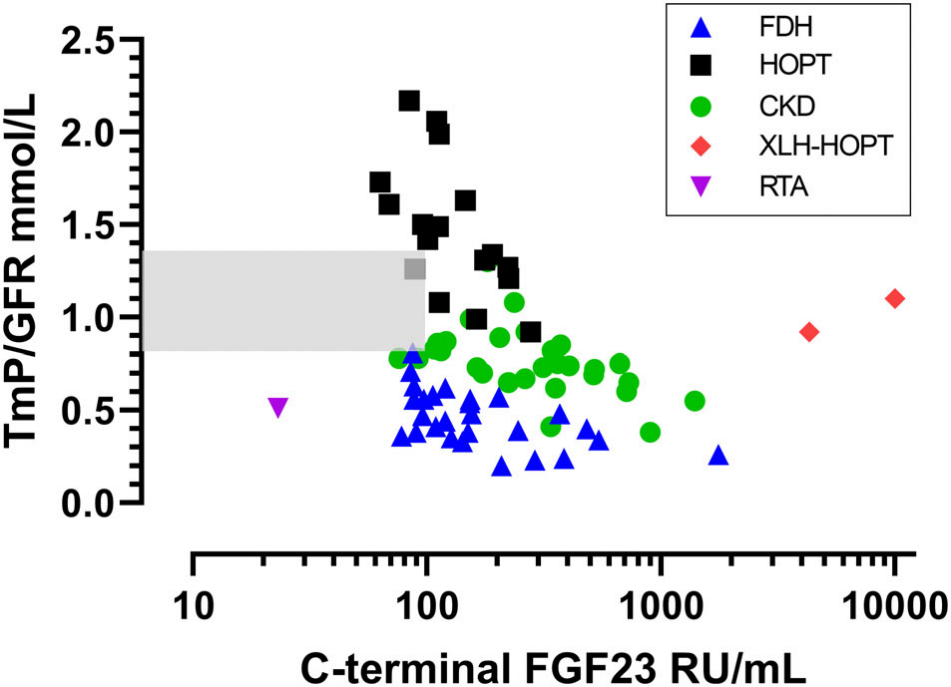
Scatterplot of TmP/GFR and log cFGF23 that includes patients with FGF23‐dependent hypophosphatemia (blue triangles), hypoparathyroidism (black squares), chronic kidney disease (green circles), the two cases of XLH‐HOPT (red diamonds), and the case of renal tubular acidosis with sensorineural deafness (purple inverted triangle). The shaded area indicates the combined reference intervals for TmP/GFR and cFGF23. There were significant correlations between TmP/GFR and log cFGF23 TmP/GFR. They were for the composite of the three groups: *r =* −.373, *p* = .001; for FDH alone: *r =* −.549, *p* = .003; for HOPT alone: *r =* −.591, *p* = .013; and for CKD alone: *r =* −.507, *p* = .004. cFGF23 = C‐terminal FGF23; CKD = chronic kidney disease; FDH = FGF23‐dependent hypophosphatemia; RTA = renal tubular acidosis; TmP/GFR = maximum renal tubular phosphate reabsorption rate per volume of glomerular filtrate; XLH‐HOPT = X‐linked hypophosphatemia with hypoparathyroidism.

**Fig 3:**
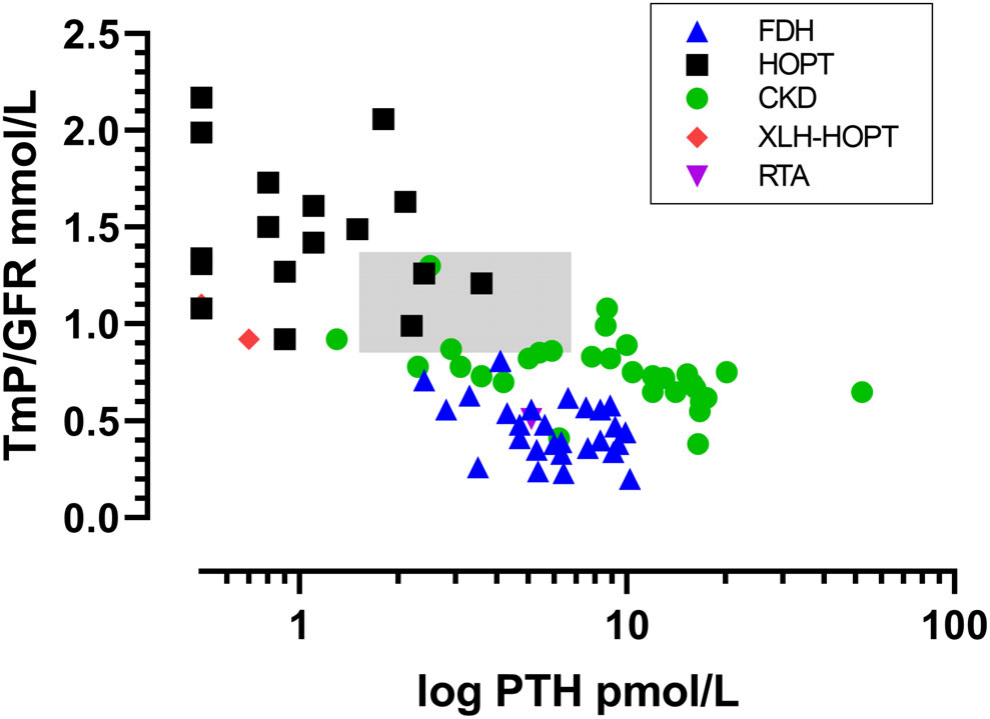
Scatterplot of TmP/GFR and log PTH that includes patients with FGF23‐dependent hypophosphatemia (blue triangles), hypoparathyroidism (black squares), CKD (green circles), the two cases of XLH‐HOPT (red diamonds), and the case of RTA with sensorineural deafness (purple inverted triangle). The shaded area represents the combined reference intervals for TmP/GFR and PTH. There were significant correlations between TmP/GFR and log PTH. They were for the composite of the three groups: *r =* −.690, *p* < .001; for FDH alone, *r =* −.394, *p* = .042; and for CKD alone: *r =* −.494, *p* = .005; but not for HOPT alone: *r =* −.207, *p* = .425. CKD = chronic kidney disease; FDH = FGF23‐dependent hypophosphatemia; RTA = renal tubular acidosis; TmP/GFR = maximum renal tubular phosphate reabsorption rate per volume of glomerular filtrate; XLH‐HOPT = X‐linked hypophosphatemia with hypoparathyroidism.

**Fig 4:**
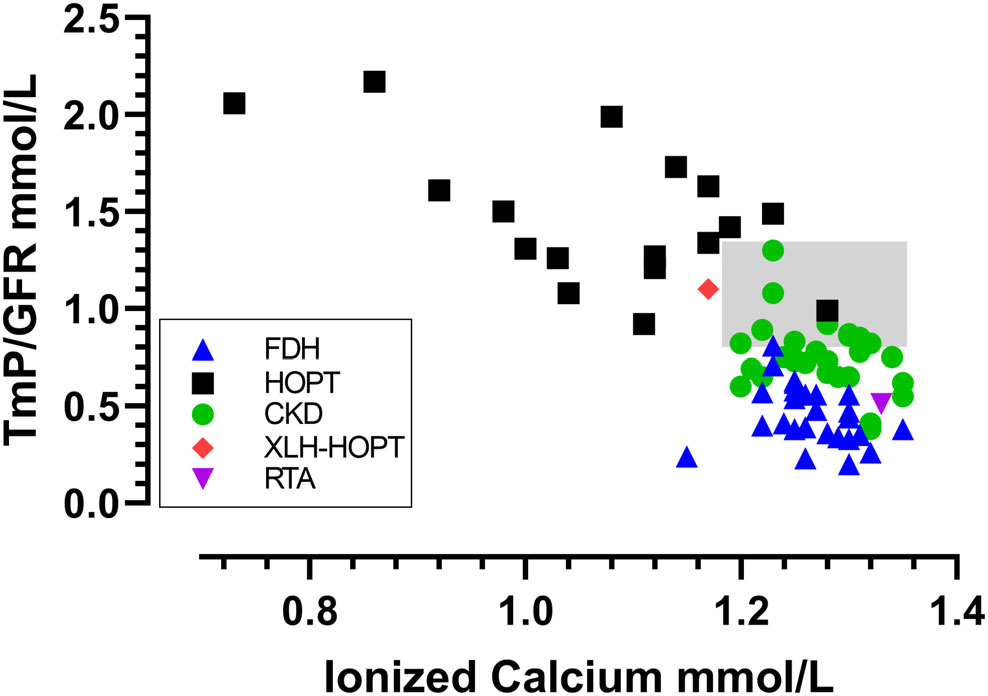
Scatterplot of TmP/GFR and ionized calcium that includes patients with FGF23‐dependent hypophosphatemia (blue triangles), hypoparathyroidism (black squares), CKD (green circles), the case 1 with XLH‐HOPT (red diamond), and the case of RTA with sensorineural deafness (purple inverted triangle). The shaded area represents the combined reference intervals for TmP/GFR and ionized calcium. There were significant correlations between TmP/GFR and ionized calcium. They were for the composite of the three groups: *r =* −.756, *p* < .001; for HOPT alone: *r =* −.569, *p* = .017; but not for FDH alone, *r =* −.254, *p* = .201; or for CKD alone: *r =* −344, *p* = .068. cFGF23 = C‐terminal FGF23; CKD = chronic kidney disease; FDH = FGF23‐dependent hypophosphatemia; RTA = renal tubular acidosis; TmP/GFR = maximum renal tubular phosphate reabsorption rate per volume of glomerular filtrate; XLH‐HOPT = X‐linked hypophosphatemia with hypoparathyroidism.

There were significant negative correlations between TmP/GFR and each of the independent variables: log cFGF23 (Fig. 2), log PTH (Fig. 3), and ionized calcium (Fig. 4). The scatterplot for TmP/GFR and log cFGF23 shows that the two cases of XLH‐HOPT are extreme outliers to the right, and that the case of RTA is an extreme outlier to the left (Fig. 2).

### Modeling analyses

Using hierarchical multiple regression, the total variance in TmP/GFR that was explained by the final model was high at 82.5%. eGFR accounted for 4.2%; log PTH, log cFGF23, and ionized calcium accounted for 77.4%; the interaction terms accounted for 3.8% with the only one achieving significance being the interaction term for log cFGF23 and log PTH. There was no evidence of multicollinearity based on low values for variance inflation factor in the final model, ranging from 1.49 to 5.71. In the final model, the three independent variables and the interaction term for log cFGF23 and log PTH were statistically significant. Beta coefficients were as follows: for log PTH, *β* = −.400, *p* < .001; for log cFGF23, *β =* −.363, *p* < .001; for ionized calcium, *β =* −.280, *p* = .008; and for the interaction term for log cFGF23 and log PTH, *β* = .203, *p* = .021. The regression analysis was repeated excluding the CKD group that yielded a high *R* ^2^ for the model summary accounting for 86.9% of the variance in TmP/GFR with significant independent effects for log cFGF23 and log PTH but not ionized calcium or any of the interactions terms.

**Fig 5:**
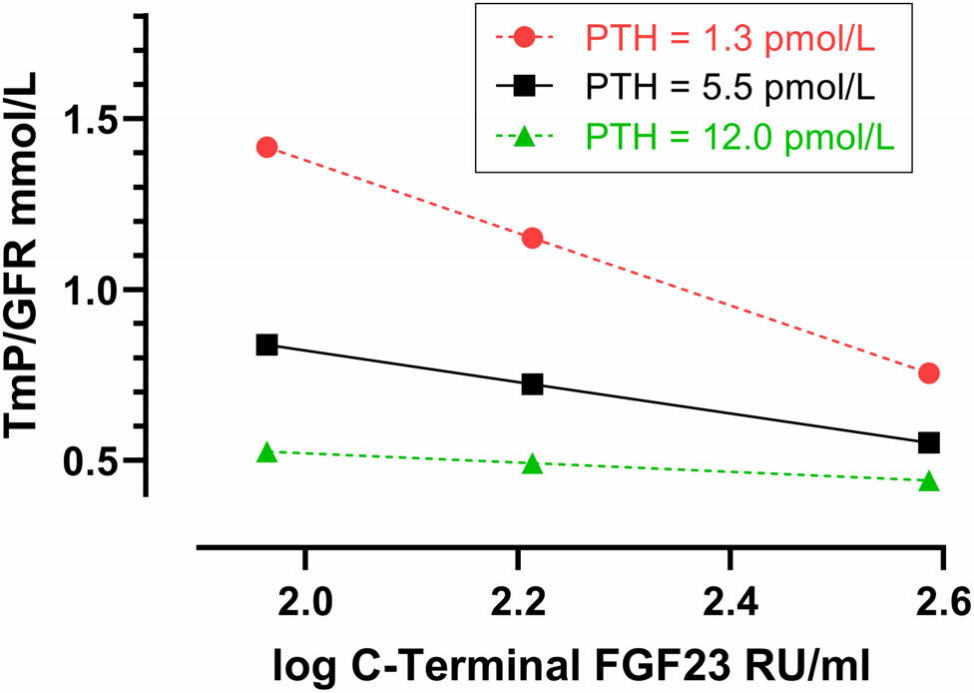
Probing the moderation effect of FGF23 on TmP/GFR by PTH, using the pick‐a‐point approach. FGF23 has a negative effect on TmP/GFR that is conditional on PTH. The effect was significant at the 16th percentile of PTH (1.3 pmol/L, *p* < .001) and at the 50th percentile of PTH (5.6 pmol/L, *p* < .001) but not at the 84th percentile of PTH (12.2 pmol/L, *p* = .284). RU = Relative unit; TmP/GFR = maximum renal tubular phosphate reabsorption rate per volume of glomerular filtrate.

**Fig 6:**
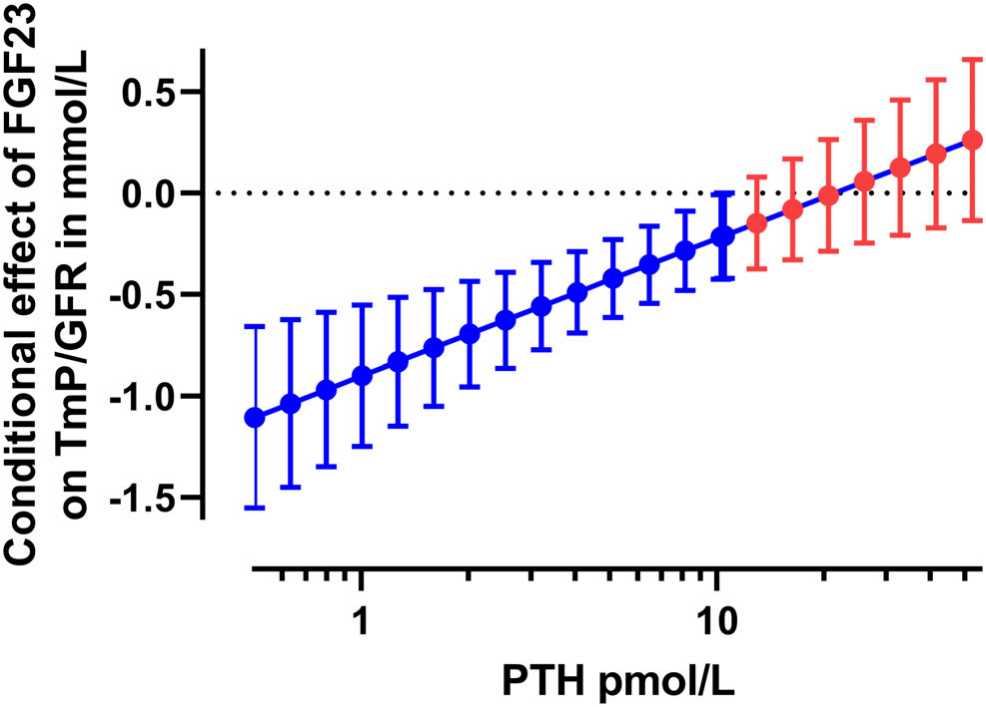
Probing the moderation effect of FGF23 on maximum renal phosphate reabsorption threshold (TmP/GFR) by PTH, using the Neyman‐Johnson technique. The point estimates and 95% confidence limits are derived across the spectrum of PTH results, ranging from 0.5 pmol/L to 52.3 pmol/L. At low PTH, the effect of FGF23 on TmP/GFR is attenuated such that a significant negative effect is seen (as depicted by blue color), but at high levels of PTH there is a nonsignificant trend for an augmented effect of FGF23 on TmP/GFR (as depicted by red color).

Similarly, using moderation analysis with PROCESS the model explained 82.4% of total variance in TmP/GFR, and the interaction term for logFGF23 and log PTH had a small but significant effect accounting for 3.4% of the variance in TmP/GFR. Regarding the conditional effects of cFGF23 on TmP/GFR at different results of PTH, significant effects were seen at the 16th percentile of 1.3 pmol/L (*p* < .001) and at the 50th percentile of 5.6 pmol/L (*p* < .001); no effect was seen at the 84th percentile of 12.2 pmol/L (*p* = .136; Fig. 5). The Johnson‐Neyman technique, in probing the moderation effect across the continuum of PTH results, showed that the effect of cFGF23 on TmP/GFR was significantly attenuated when PTH was low, and that the effect of cFGF23 on TmP/GFR was trending towards being augmented when PTH was high (Fig. 6).

### Intervention study

**Fig 7:**
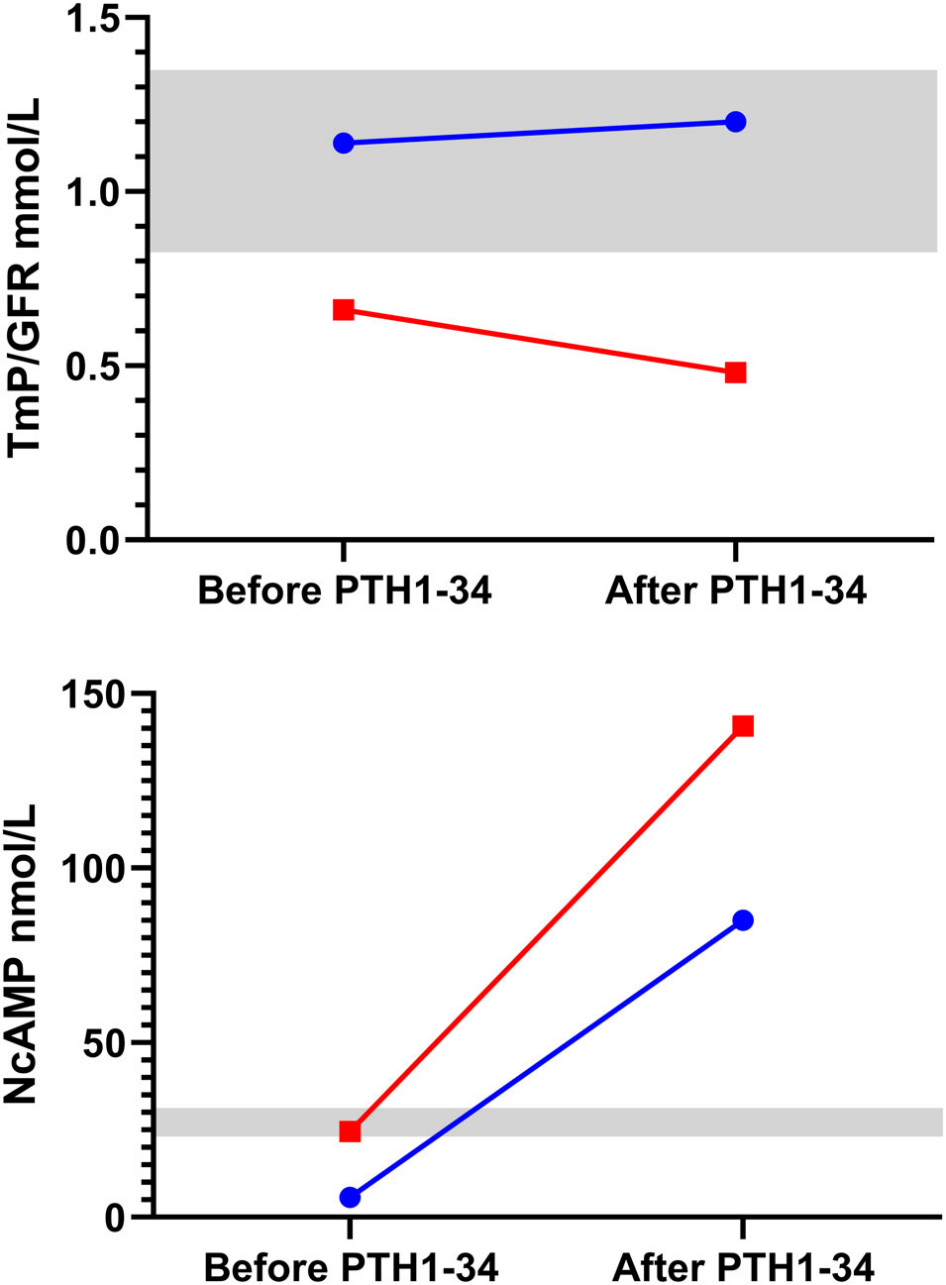
Response to rhPTH1‐34 of TmP/GFR (upper panel) and of NcAMP (lower panel) in the case of XLH‐HOPT before and after first injection (blue circles), and before and after the 28th daily injection (red squares). Reference intervals are represented by the shaded areas. NcAMP = nephrogenous cyclic adenosine monophosphate; TmP/GFR = maximum renal tubular phosphate reabsorption rate per volume of glomerular filtrate.

After the first injection of rhPTH1‐34 in case 1 with XLH‐HOPT, TmP/GFR remained unchanged within the reference interval, but NcAMP increased from very low baseline at 5.7 nmol/L to 85.1 nmol/L after the first dose (Table 3, Fig. 7). After 28 days of PTH1‐34, the morning fasting TmP/GFR prior to the final injection was low at 0.66 mmol/L; after the final injection, it was even lower at 0.48 mmol/L. Fasting NcAMP was higher than the baseline measurement at 24.5 nmol/L; it increased to 140.7 nmol/L after the final injection. iFGF23 declined by 2250 pg/mL but it was still markedly elevated at 9650 pg/mL, and there were no changes in cFGF23 (Table 3). Soluble α‐Klotho was low and remained low. Fasting ionized calcium was unchanged after 28 days; it increased after the final injection but was still low. After 28 days, all bone turnover markers had increased markedly from baseline, ranging from 114% for PINP to 287% for uNTX. There were no changes in vitamin D metabolites or in the vitamin D metabolite ratios.

**Table 3:** Response to rhPTH1‐34 20 μg Daily for 28 Days in Case 1 with X‐Linked Hypophosphatemia with Hypoparathyroidism

Variables	Pre‐day 1	Post‐day 1	Pre‐day 28	Post‐day 28	Reference intervals
TmP/GFR, mmol/L	1.14	1.20	0.66	0.48	0.81, 1.35
NcAMP, nmol/L	5.7	85.1	24.5	140.7	22.2, 30.4
cFGF23, RU/L	6700	7300	6750	7050	<100
iFGF23, ng/L	11,900	12,200	10,400	9650	10, 50
α‐Klotho, ng/L	346.4	408.7	407.7	408.3	505, 1175
Ionized calcium, mmol/L	1.08	1.07	1.07	1.16	1.19, 1.35
PTH, pmol/L	<0.6	<0.6	<0.6	<0.6	1.6, 6.9
PINP, μg/L	41.5	41.4	89.8	89.2	22, 96
OC(1–43), μg/L	15.2	14.3	32.7	33.9	11, 43
Bone ALP, μg/L	5.5	8.8	16	15.1	2.9, 20.9
CTX, μg/L	0.370	0.437	0.641	1.060	0.016, 0.584
uNTX/Cr, nmol BCE/mmol Cr	9.9	3.6	38.2	38.3	14, 74
uCa/Cr mmol/mmol	0.41	0.47	0.65	0.33	0.07, 0.41
25OHD, nmol/L	98	113	84	90	30, 125
24,25(OH)_2_D, nmol/L	3.5	3.6	2.8	3.5	1.0, 13.0
1,25(OH)_2_D, pmol/L	57	63	53	51	55, 139
25OHD:24,25(OH)_2_D	28	31	30	26	7, 25
1,25(OH)_2_D:24,25(OH)_2_D	16	18	19	15	9, 132

1,25(OH)_2_D = 1,25‐hydroxyvitamin D; 25OHD = 25‐hydroxyvitamin D; ALP = alkaline phosphatase; BAP = bone‐specific alkaline phosphatase; BCE = bone collagen equivalent; cFGF23 = C‐terminal FGF23; Ca = calcium; Cr = creatinine; CTX = C‐terminal telopeptide of type I collagen; iFGF23 = intact FGF23; OC(1–43) = N‐mid fragment osteocalcin; PINP = procollagen type I N propeptide; NcAMP = nephrogenous cyclic adenosine monophosphate; NTX = N‐terminal telopeptides of type I collagen; RU = relative unit; TmP/GFR = maximum renal tubular phosphate reabsorption rate per volume of glomerular filtrate; uNTX = urine N‐terminal telopeptides of type I collagen.

## Discussion

This clinical study supports the premise, as reviewed in the Introduction, that there are independent effects of FGF23, PTH, and calcium on TmP/GFR. In both the total group and each of the three groups, log cFGF23 correlated negatively with TmP/GFR. Similarly, in both the total group and in two of the three groups (FDH and CKD), PTH correlated negatively with TmP/GFR. Ionized calcium correlated negatively with TmP/GFR in the total group and in HOPT. Hierarchical multiple regression analysis and moderation analysis by PROCESS established negative independent effects on TmP/GFR of all three variables (log cFGF23, log PTH, and ionized calcium) and found a small but significant effect of the interaction term for PTH and cFGF23. Probing analysis showed that the effect varied across the continuum of PTH results after adjusting for ionized calcium: At low PTH, the effect of cFGF23 on TmP/GFR was attenuated; at high PTH, the effect of cFGF23 on TmP/GFR was trending towards being augmented. In the patient with XLH‐HOPT, who received a 28‐day course of rhPTH1‐34, there was a substantial lowering of TmP/GFR to within the FDH range that was accompanied by renal tubular response to PTH as manifested by the increase in NcAMP. This change in TmP/GFR was achieved without any alteration in CKD status and minimal change in ionized calcium. Contemporaneous to this TmP/GFR and NcAMP response, there was a considerable increase in bone turnover markers, but there was no effect on α‐Klotho or on vitamin D metabolism. Therefore, our findings indicate that the lowering effect of FGF23 excesson TmP/GFR is moderated by PTH.

We chose TmP/GFR because it is the best measure of renal phosphate handling in the steady state.^(^ ^22^ ^)^ TmP/GFR is superior to the fractional tubular reabsorption of phosphate and to its corollary, the fractional phosphate excretion, because it is independent of both GFR—at least down to a GFR of 40 mL/min—and the net inflow of phosphate from the intestine, bone, and extracellular tissues.^(^ ^22^ ^)^ Using either a nomogram or an equation, the TmP/GFR is readily calculated from measurements of phosphate and creatinine in paired fasting samples of blood and urine.^(^ ^21^ ^)^ Although both PTH and FGF23 are characterized as phosphaturic hormones, which is a correct descriptor with respect to their acute effect following parenteral administration, it is the steady state lowering of TmP/GFR that results in chronic hypophosphatemia and mineralization defect in bone.^(^ ^3^, ^5^ ^)^

In the literature, there are contradictory findings in XLH about the effect of serum calcium on renal phosphate wasting. High‐dose calcium infusion over 6 to 8 hours in XLH lessened renal phosphate wasting, as evidenced by a marked increase in tubular reabsorption of phosphate.^(^ ^19^ ^)^ By contrast, in a case of coincidental XLH and HOPT, the TmP/GFR in the untreated state was high at 2.1 mmol/L; after restoring serum calcium to normal with activated vitamin D, TmP/GFR was low at 0.58 mmol/L.^(^ ^17^ ^)^ In exploring the effect of ionized calcium on TmP/GFR, our findings are consistent with the contradictory effects reported in the literature about the effect of calcium on renal phosphate wasting. Serum ionized calcium negatively correlated with TmP/GFR, similar to both PTH and cFGF23. Modeling analysis found that serum ionized calcium had a significant negative effect on TmP/GFR; this effect was independent of both PTH and FGF23, but the beta coefficient in the regression model was lower. This independent negative effect is consistent with findings in the case report of XLH and coincidental HOPT.^(^ ^17^ ^)^ The mechanism of this direct effect is not known. Partial correlation analysis found that PTH attenuated the effect of ionized calcium on TmP/GFR, which is consistent with the finding that high‐dose calcium infusion over 6 to 8 hours decreased renal phosphate excretion by PTH suppression.^(^ ^19^ ^)^ Thus, extracellular calcium has both a direct effect that increases renal phosphate wasting as well as an opposite, but indirect, effect that decreases renal phosphate wasting via PTH suppression. Separating the effects of calcium and PTH on TmP/GFR might be possible in the study of disorders with mutations in the calcium‐sensing receptor.

A confounding factor in our study is that both cases of XLH‐HOPT had CKD unlike the cases in the FDH group (Table 1).^(^ ^13^, ^14^ ^)^ Given that both FGF23 and serum phosphate increase with the decline in eGFR and given that there is dysregulated production of FGF23 in XLH, it is possible that CKD might explain both the TmP/GFR results and the FGF23 results in the two cases of XLH‐HOPT. For this reason, a small group of patients with stable CKD was studied. As expected in this CKD group, cFGF23 had a negative correlation with eGFR.^(^ ^29^ ^)^ That TmP/GFR was low in 63% of patients—with all but one patient having normal serum phosphate—is consistent with the combined compensatory effect of elevated cFGF23 and secondary hyperparathyroidism in this group with stable CKD. Regarding the two cases with XLH‐HOPT, cFGF23 results were much higher than expected for eGFR, whereas TmP/GFR results were toward the upper end of that seen in the CKD group. That TmP/GFR is higher than expected for the degree of CKD despite marked elevation in both cFGF23 and iFGF23 is best explained by the moderation effects of both PTH deficiency and hypocalcemia. The patient with congenital RTA with sensorineural deafness had low TmP/GFR with low‐normal cFGF23 and low iFGF23, despite having CKD. This case suggests that hypophosphatemia in the setting of CKD is associated with appropriate physiological suppression of FGF23 production, superseding the tendency for FGF23 to increase in CKD.

The dependence of FGF23 on both PTH and calcium for its full effect on TmP/GFR is supported by both clinical observations and by animal studies. In the original description of XLH by Albright and colleagues in 1937, which was initially termed vitamin‐D–resistant rickets, excision of a hyperplastic parathyroid gland was followed by transient hypoparathyroidism and normalization of serum phosphate.^(^ ^15^ ^)^ In the era prior to FGF23 measurement, a 1969 case report of hypophosphatemic bone disease noted that total parathyroidectomy corrected renal phosphate wasting and osteomalacia^(^ ^16^ ^)^; and a case report from 1985 of XLH and coincidental idiopathic HOPT noted an elevated TmP/GFR as summarized above.^(^ ^17^ ^)^ A 2013 case report of TIO noted that HOPT, following total parathyroidectomy for tertiary hyperparathyroidism, normalized TmP/GFR and cured osteomalacia on histomorphometric findings despite persistently high cFGF23.^(^ ^12^ ^)^ Conversely, in a study of patients with HOPT in whom cFGF23 and serum phosphate were measured, the authors concluded that high FGF23 was not sufficient to avert hyperphosphatemia caused by HOPT.^(^ ^9^ ^)^ A similar finding was evident in our HOPT group. In addition, we recently reported a case of anorexia nervosa in whom TmP/GFR was elevated despite high cFGF23 during an episode of transient functional hypoparathyroidism.^(^ ^30^ ^)^

In a study of parathyroidectomized rats, injection of FGF23 lowered serum phosphate. However, after FGF23 injection the serum phosphate was higher in the parathyroidectomized rats compared with sham‐operated rats.^(^ ^1^ ^)^ In the *Hyp* mouse, which is an animal model for XLH, a study of *Pth* knockout in the Hyp hemizygous mice (*Pth* ^*−*^ */* ^*−*^, *Hyp/Y)* showed that serum phosphate levels were similar to *Pth*‐null‐only mice despite marked elevation in iFGF23 in the Hyp hemizygotes.^(^ ^31^ ^)^ We have made an equivalent observation in our two cases of XLH‐HOPT, whereby TmP/GFR was in the range of HOPT cases rather than XLH cases. In addition, administration of bovine PTH1‐34 to the *Pth*‐null *Hyp* mice lowered serum phosphate, mirroring the findings seen in our rhPTH1‐34 trial.

We sought to explore the independent effects on TmP/GFR in a cross‐sectional clinical study by modeling analysis to determine whether PTH alters the effect of FGF23 excess on TmP/GFR. If PTH alters the effect of FGF23 excess, then the magnitude of the causal effect of FGF23 on TmP/GFR could be lessened by hypoparathyroidism or it could be augmented by hyperparathyroidism. Using the traditional statistical approach of hierarchical linear regression analysis, there were significant independent negative effects of PTH, cFGF23, and ionized calcium on TmP/GFR and a small but significant effect of the interaction term for PTH and cFGF23. Moderation analysis with PROCESS probed this effect of FGF23 on TmP/GFR across the PTH continuum; it showed lessening of the effect when PTH was low and trending towards augmenting the effect when PTH was high. In keeping with this effect, case 1 with XLH‐HOPT and near‐normal ionized calcium on activated vitamin D showed that PTH deficiency attenuated the effect of FGF23 on TmP/GFR to the degree that TmP/GFR was no longer lowered and bone turnover markers were within the reference intervals (Table 1).^(^ ^14^ ^)^

We do not have an explanation for two findings: the degree of elevation in FGF23, both C‐terminal and intact; and how the effect of FGF23 excess on TmP/GFR is altered by PTH. Regarding the first issue, the sensing mechanism for stimulating FGF23 in response to phosphate is unknown. In mice, high phosphate intake did not enhance expression of *Fgf23* but enhanced the expression of *Galnt3*, a gene that prevents cleavage of iFGF23.^(^ ^32^ ^)^ Better understanding of this regulation could explain the marked elevation in FGF23 in our two cases of XLH‐HOPT. Regarding the second issue, the effect is likely to be at the level of the proximal renal tubule. Although both FGF23 and PTH inhibit reabsorption of phosphate on the luminal border, they are known to act through different receptors on the basal surface and by different pathways within the cell.^(^ ^33^ ^)^ As evidenced by the rise in nephrogenous cAMP following administration of rhPTH1‐34 in case 1 with XLH‐HOPT, there was an immediate renal tubular response after the first injection and an even more robust response after the final injection. There was no change in TmP/GFR after the first injection but TmP/GFR was low prior to final injection and was even lower after the final injection. Ionized calcium remained low throughout but there was an increase after the final injection. The decline in TmP/GFR reflects mainly a direct effect of rhPTH1‐34 but also may reflect a direct effect of calcium. Over the 28 days, there were also substantive increases in all the bone turnover markers. Soluble α‐Klotho was low prior to the first rhPTH1‐34 injection and remained low throughout the 28 days, the most likely explanation being the expected decline in soluble α‐Klotho that is seen in CKD.^(^ ^26^ ^)^ There was a small decline in 1,25(OH)_2_D, which was low‐normal at the outset. There were no changes in the vitamin D metabolite ratios, which are considered surrogate markers of the hydroxylation enzymes CYP27B1 and CYP24A1.^(^ ^27^, ^34^ ^)^ FGF23 reduces CYP27B1 activity and increases CYP24A1 activity leading to a reduction in 1,25(OH)_2_D in favor of increased production of 24,25(OH)_2_D, whereas PTH increases CYP27B1 activity.

This study has several limitations. First, selection bias could contribute to the findings of the modeling analysis being spurious. Given our small sample size, the possibility of reaching significance for the modeling analysis was enhanced using a composite group having a wide range of results for four main variables (TmP/GFR, cFGF23, PTH, and ionized calcium); the separateness of the three groups was confirmed by discriminant function analysis. That the model explained 82.5% of the variance in TmP/GFR reflects the broad spectrum of results, such that the composite nearly forms a continuum along the full range of results for the different tests. It is likely that the proportionate effects of each independent variable (FGF23, PTH, and ionized calcium) on TmP/GFR vary with each disorder of renal phosphate handling. These independent effects need to be validated in larger homogenous groups. Also, because our conclusions are based on cFGF23, which includes both iFGF23 and fragments, measurement of iFGF23 would be needed to validate our findings. A related limitation is the suggestion that excess cFGF23, as seen in our two cases, could inhibit the effect of iFGF23.^(^ ^35^ ^)^ Second, confounding could apply in that a number of unmeasured factors may have explained the independent effects. The effect of activated vitamin D products was not controlled for in our modeling analysis. Other factors that were not studied could contribute to changes in TmP/GFR such as: variations in growth hormone or cortisol,^(^ ^36^, ^37^ ^)^ iron deficiency,^(^ ^38^ ^)^ iron infusions,^(^ ^39^, ^40^ ^)^ and nicotinamide phosphoribosyltransferase.^(^ ^41^ ^)^ Third, physiologic fluctuation of TmP/GFR may explain the fall in TmP/GFR after PTH1‐34, but the degree of lowering at 58%, in conjunction with changes in both NcAMP and bone turnover markers, would be difficult to explain as a chance occurrence.

This moderating effect of PTH on FGF23‐mediated lowering of TmP/GFR has clinical implications. Although we have highlighted the clinical significance in both XLH and TIO regarding the rare circumstance of total parathyroidectomy, a more common clinical implication is hyperparathyroidism in XLH.^(^ ^11^, ^18^ ^)^ The need for phosphate supplementation as treatment for XLH is the principal cause of hyperparathyroidism.^(^ ^42^ ^)^ In adults with XLH, secondary hyperparathyroidism is common, ranging in prevalence from 15% to 67%.^(^ ^14^, ^18^, ^43^, ^44^ ^)^ In two recent large surveys of XLH, tertiary hyperparathyroidism was evident in 10% to 17% of cases.^(^ ^18^, ^44^ ^)^ Hyperparathyroidism, as a complication of treating XLH, should diminish given the success of blocking FGF23 activity with burosumab, which obviates the need for phosphate supplements in both children and adults.^(^ ^45^, ^46^ ^)^ There are two other clinical circumstances where hyperparathyroidism in conjunction with FGF23 excess could exacerbate hypophosphatemia. After renal transplantation, FGF23‐mediated hypophosphatemia and hyperparathyroidism may be coincident.^(^ ^5^ ^)^ After intravenous iron infusion, hypophosphatemia caused by FGF23‐mediated phosphaturia is commonly observed.^(^ ^39^, ^40^, ^47^ ^)^ This lowering of TmP/GFR after iron infusion is likely to be augmented if patients have secondary hyperparathyroidism. Finally, regarding therapy for hyperparathyroidism in the setting of FGF23 excess, it has been reported in two cases of TIO that cinacalcet countered the effect of FGF23 on renal phosphate wasting by lowering PTH; however, it has never been tested in a clinical trial and there is concern about hypercalciuria and acute kidney injury.^(^ ^10^ ^)^

In conclusion, PTH alters renal phosphate handling in clinical states of FGF23 excess. Hypoparathyroidism attenuates the TmP/GFR lowering effect of FGF23 excess, such that hypophosphatemic bone disease is ameliorated. Hyperparathyroidism augments the TmP/GFR lowering effect of FGF23 excess. The mechanism by which PTH alters the effect of FGF23 excess on TmP/GFR warrants investigation.

## Disclosures

MJM received fees for lectures or advice from Amgen, Clonmel Healthcare, Mylan, Pharmacosmos, and UCB. RKC received travel support from Amgen. None of the other authors have any conflicts of interest.

## Author Contributions

**Malachi McKenna:** Conceptualization; data curation; formal analysis; investigation; software; validation; writing‐original draft; writing‐review and editing. **Rachel Crowley:** Conceptualization; writing‐review and editing. **Patrick Twomey:** Methodology; writing‐review and editing. **Mark Kilbane:** Conceptualization; data curation; methodology; validation; writing‐review and editing.

### Peer Review

The peer review history for this article is available at https://publons.com/publon/10.1002/jbm4.10437.

## References

[1] Shimada T , Hasegawa H , Yamazaki Y , et al. FGF‐23 is a potent regulator of vitamin D metabolism and phosphate homeostasis. J Bone Miner Res. 2004;19(3):429–35. 15040831 10.1359/JBMR.0301264

[2] Gattineni J , Bates C , Twombley K , et al. FGF23 decreases renal NaPi‐2a and NaPi‐2c expression and induces hypophosphatemia in vivo predominantly via FGF receptor 1. Am J Physiol Renal Physiol. 2009;297(2):F282–91. 19515808 10.1152/ajprenal.90742.2008 PMC2724258

[3] Marcucci G , Masi L , Ferrari S , et al. Phosphate wasting disorders in adults. Osteoporos Int. 2018;29(11):2369–87. 30014155 10.1007/s00198-018-4618-2

[4] Insogna KL , Rauch F , Kamenický P , et al. Burosumab improved histomorphometric measures of osteomalacia in adults with X‐linked hypophosphatemia: a phase 3, single‐arm, international trial. J Bone Miner Res. 2019;34(12):2183–91. 31369697 10.1002/jbmr.3843 PMC6916280

[5] Imel EA , Econs MJ . Approach to the hypophosphatemic patient. J Clin Endocrinol Metab. 2012;97(3):696–706. 22392950 10.1210/jc.2011-1319 PMC3319220

[6] Stewart AF , Horst R , Deftos LJ , Cadman EC , Lang R , Broadus AE . Biochemical evaluation of patients with cancer‐associated hypercalcemia: evidence for humoral and nonhumoral groups. N Engl J Med. 1980;303(24):1377–83. 6253785 10.1056/NEJM198012113032401

[7] Bijvoet OL , Morgan DB , Fourman P . The assessment of phosphate reabsorption. Clin Chim Acta. 1969;26(1):15–24. 5356592 10.1016/0009-8981(69)90280-0

[8] Parfitt AM , Rao DS , Stanciu J , Villanueva AR , Kleerekoper M , Frame B . Irreversible bone loss in osteomalacia. Comparison of radial photon absorptiometry with iliac bone histomorphometry during treatment. J Clin Invest. 1985;76(6):2403–12. 4077986 10.1172/JCI112253 PMC424391

[9] Gupta A , Winer K , Econs MJ , Marx SJ , Collins MT . FGF‐23 is elevated by chronic hyperphosphatemia. J Clin Endocrinol Metab. 2004;89(9):4489–92. 15356053 10.1210/jc.2004-0724

[10] Geller JL , Khosravi A , Kelly MH , Riminucci M , Adams JS , Collins MT . Cinacalcet in the management of tumor‐induced osteomalacia. J Bone Miner Res. 2007;22(6):931–7. 17352646 10.1359/jbmr.070304

[11] Carpenter TO , Olear EA , Zhang JH , et al. Effect of paricalcitol on circulating parathyroid hormone in X‐linked hypophosphatemia: a randomized, double‐blind, placebo‐controlled study. J Clin Endocrinol Metab. 2014;99(9):3103–11. 25029424 10.1210/jc.2014-2017 PMC4154090

[12] Bhadada SK , Palnitkar S , Qiu S , Parikh N , Talpos GB , Rao SD . Deliberate total parathyroidectomy: a potentially novel therapy for tumor‐induced hypophosphatemic osteomalacia. J Clin Endocrinol Metab. 2013;98(11):4273–8. 23956343 10.1210/jc.2013-2705

[13] Crowley RK , Kilbane M , King TF , Morrin M , O'Keane M , McKenna MJ . Hungry bone syndrome and normalisation of renal phosphorus threshold after total parathyroidectomy for tertiary hyperparathyroidism in X‐linked hypophosphataemia: a case report. J Med Case Rep. 2014;8(1):84. 24594262 10.1186/1752-1947-8-84 PMC3946034

[14] McKenna MJ , Martin‐Grace J , Crowley R , Twomey PJ , Kilbane MT . Congenital hypophosphataemia in adults: determinants of bone turnover markers and amelioration of renal phosphate wasting following total parathyroidectomy. J Bone Miner Metab. 2019;37(4):685–93. 30238432 10.1007/s00774-018-0957-5

[15] Albright F , Butler AM , Bloomberg E . Rickets resistant to vitamin D therapy. Am J Dis Child. 1937;54(3):529–47.

[16] Riggs BL , Sprague RG , Jowsey J , Maher FT . Adult‐onset vitamin‐D‐resistant hypophosphatemic osteomalacia. Effect of total parathyroidectomy. N Engl J Med. 1969;281(14):762–6. 5807923 10.1056/NEJM196910022811404

[17] Lyles KW , Burkes EJ Jr , McNamara CR , Harrelson JM , Pickett JP , Drezner MK . The concurrence of hypoparathyroidism provides new insights to the pathophysiology of X‐linked hypophosphatemic rickets. J Clin Endocrinol Metab. 1985;60(4):711–7. 4038714 10.1210/jcem-60-4-711

[18] Lecoq AL , Chaumet‐Riffaud P , Blanchard A , et al. Hyperparathyroidism in patients with X‐linked hypophosphatemia. J Bone Miner Res. 2020;35(7):1263–73. 32101626 10.1002/jbmr.3992

[19] Falls WF Jr , Carter NW , Rector FC Jr , Seldin DW . Familial vitamin D‐resistant rickets. Study of six cases with evaluation of the pathogenetic role of secondary hyperparathyroidism. Ann Intern Med. 1968;68(3):553–60. 5643679 10.7326/0003-4819-68-3-553

[20] Glorieux F , Scriver CR . Loss of a parathyroid hormone‐sensitive component of phosphate transport in X‐linked hypophosphatemia. Science. 1972;175(4025):997–1000. 4333173 10.1126/science.175.4025.997

[21] Walton RJ , Bijvoet OL . Nomogram for derivation of renal threshold phosphate concentration. Lancet. 1975;2(7929):309–10. 50513 10.1016/s0140-6736(75)92736-1

[22] Bijvoet OL . Relation of plasma phosphate concentration to renal tubular reabsorption of phosphate. Clin Sci. 1969;37(1):23–36. 5822530

[23] Horowitz GL , Altaie S , Boyd JC . Defining, establishing and verifying reference intervals in the clinical laboratory; approved guideline. 3rd ed Clinical Laboratory Standards Institute: Wayne, PA; 2010.

[24] Wu AHB . Tietz clinical guide to laboratory tests. 4th ed Saunders/Elsevier: St. Louis, MO; 2006.

[25] Babka JC , Bower RH , Sode J . Nephrogenous cyclic AMP levels in primary hyperparathyroidism. Arch Intern Med. 1976;136(10):1140–4. 184749

[26] Rotondi S , Pasquali M , Tartaglione L , et al. Soluble alpha‐Klotho serum levels in chronic kidney disease. Int J Endocrinol. 2015;2015:872193. 25873958 10.1155/2015/872193 PMC4383388

[27] Tang JCY , Jackson S , Walsh NP , Greeves J , Fraser WD , Bioanalytical Facility Team . The dynamic relationships between the active and catabolic vitamin D metabolites, their ratios, and associations with PTH. Sci Rep. 2019;9(1):6974. 31061425 10.1038/s41598-019-43462-6 PMC6502854

[28] Hayes AF . Introduction to mediation, moderation, and conditional process analysis. New York: Guilford Press; 2013.

[29] Gutierrez O , Isakova T , Rhee E , et al. Fibroblast growth factor‐23 mitigates hyperphosphatemia but accentuates calcitriol deficiency in chronic kidney disease. J Am Soc Nephrol. 2005;16(7):2205–15. 15917335 10.1681/ASN.2005010052

[30] Kilbane MT , Crowley RK , Twomey PJ , Maher C , McKenna MJ . Anorexia nervosa with markedly high bone turnover and hyperphosphatemia during refeeding rectified by denosumab. Osteoporos Int. 2020;31(7):1395–8. 31975181 10.1007/s00198-020-05307-1

[31] Bai X , Miao D , Goltzman D , Karaplis AC . Early lethality in Hyp mice with targeted deletion of Pth gene. Endocrinology. 2007;148(10):4974–83. 17615144 10.1210/en.2007-0243

[32] Takashi Y , Kosako H , Sawatsubashi S , et al. Activation of unliganded FGF receptor by extracellular phosphate potentiates proteolytic protection of FGF23 by its O‐glycosylation. Proc Natl Acad Sci U S A. 2019;116(23):11418–27. 31097591 10.1073/pnas.1815166116 PMC6561303

[33] Erben RG , Andrukhova O . FGF23 regulation of renal tubular solute transport. Curr Opin Nephrol Hypertens. 2015;24(5):450–6. 26125643 10.1097/MNH.0000000000000145

[34] Cavalier E , Huyghebaert L , Rousselle O , et al. Simultaneous measurement of 25(OH)‐vitamin D and 24,25(OH)2‐vitamin D to define cut‐offs for CYP24A1 mutation and vitamin D deficiency in a population of 1200 young subjects. Clin Chem Lab Med. 2020;58(2):197–201. 31804956 10.1515/cclm-2019-0996

[35] Boyce AM , Bhattacharyya N , Collins MT . Fibrous dysplasia and fibroblast growth factor‐23 regulation. Curr Osteoporos Rep. 2013;11(2):65–71. 23532406 10.1007/s11914-013-0144-5 PMC3669677

[36] Lieberman SA , Holloway L , Marcus R , Hoffman AR . Interactions of growth hormone and parathyroid hormone in renal phosphate, calcium, and calcitriol metabolism and bone remodeling in postmenopausal women. J Bone Miner Res. 1994;9(11):1723–8. 7863823 10.1002/jbmr.5650091108

[37] Takuwa Y , Yamamoto M , Matsumoto T , Hata K , Ogata E . Hyperphosphatemia after surgical correction of hypercortisolism in patients with Cushing's syndrome. Miner Electrolyte Metab. 1986;12(2):119–24. 3960015

[38] Imel EA , Liu Z , Coffman M , Acton D , Mehta R , Econs MJ . Oral iron replacement normalizes fibroblast growth factor 23 in iron‐deficient patients with autosomal dominant hypophosphatemic rickets. J Bone Miner Res. 2019;35(2):231–8. 31652009 10.1002/jbmr.3878 PMC7333537

[39] Detlie TE , Lindstrom JC , Jahnsen ME , et al. Incidence of hypophosphatemia in patients with inflammatory bowel disease treated with ferric carboxymaltose or iron isomaltoside. Aliment Pharmacol Ther. 2019;50(4):397–406. 31264261 10.1111/apt.15386

[40] Wolf M , Rubin J , Achebe M , et al. Effects of iron isomaltoside vs ferric carboxymaltose on hypophosphatemia in iron‐deficiency anemia: two randomized clinical trials. JAMA. 2020;323(5):432–43. 32016310 10.1001/jama.2019.22450 PMC7042864

[41] Miyagawa A , Tatsumi S , Takahama W , et al. The sodium phosphate cotransporter family and nicotinamide phosphoribosyltransferase contribute to the daily oscillation of plasma inorganic phosphate concentration. Kidney Int. 2018;93(5):1073–85. 29398136 10.1016/j.kint.2017.11.022

[42] Carpenter TO , Imel EA , Holm IA , Jan de Beur SM , Insogna KL . A clinician's guide to X‐linked hypophosphatemia. J Bone Miner Res. 2011;26(7):1381–8. 21538511 10.1002/jbmr.340 PMC3157040

[43] Shanbhogue VV , Hansen S , Jorgensen NR , Beck‐Nielsen SS . Impact of conventional medical therapy on bone mineral density and bone turnover in adult patients with X‐linked hypophosphatemia: a 6‐year prospective cohort study. Calcif Tissue Int. 2018;102(3):321–8. 29143140 10.1007/s00223-017-0363-3

[44] DeLacey S , Liu Z , Broyles A , et al. Hyperparathyroidism and parathyroidectomy in X‐linked hypophosphatemia patients. Bone. 2019;127:386–92. 31276850 10.1016/j.bone.2019.06.025 PMC6836672

[45] Carpenter TO , Whyte MP , Imel EA , et al. Burosumab therapy in children with X‐linked hypophosphatemia. N Engl J Med. 2018;378(21):1987–98. 29791829 10.1056/NEJMoa1714641

[46] Insogna KL , Briot K , Imel EA , et al. A randomized, double‐blind, placebo‐controlled, phase 3 trial evaluating efficacy of burosumab, an anti‐FGF23 antibody, in adults with X‐linked hypophosphatemia: week 24 primary analysis. J Bone Miner Res. 2018;33(8):1383–93. 29947083 10.1002/jbmr.3475

[47] Posod A , Schaefer B , Mueller T , Zoller H , Kiechl‐Kohlendorfer U . Hypophosphatemia in children treated with ferric carboxymaltose. Acta Paediatr. 2020;109(7):1491–2. 31955454 10.1111/apa.15178 PMC7318602

